# Bone marrow produces sufficient alloreactive natural killer (NK) cells in vivo to cure mice from subcutaneously and intravascularly injected 4T1 breast cancer

**DOI:** 10.1007/s10549-016-4067-6

**Published:** 2016-12-03

**Authors:** Michel van Gelder, Ariane Vanclée, Catharina H. M. J. van Elssen, Pierre Hupperets, Lotte Wieten, Gerard M. Bos

**Affiliations:** 1 Department of Internal Medicine, Maastricht University Medical Center, Postbus 5800, 6202 AZ Maastricht, The Netherlands; 2 Department of Transplantation Immunology, Maastricht University Medical Center, Postbus 5800, 6202 AZ Maastricht, The Netherlands

**Keywords:** 4T1, Breast cancer, Immunotherapy, Alloreactive NK cell, Hematopoietic stem cell transplantation

## Abstract

**Purpose:**

Administration of 5 million alloreactive natural killer (NK) cells after low-dose chemo-irradiation cured mice of 4T1 breast cancer, supposedly dose dependent. We now explored the efficacy of bone marrow as alternative in vivo source of NK cells for anti-breast cancer treatment, as methods for in vitro clinical scale NK cell expansion are still in developmental phases.

**Methods:**

Progression-free survival (PFS) after treatment with different doses of spleen-derived alloreactive NK cells to 4T1-bearing Balb/c mice was measured to determine a dose–response relation. The potential of bone marrow as source of alloreactive NK cells was explored using MHC-mismatched mice as recipients of 4T1. Chemo-irradiation consisted of 2× 2 Gy total body irradiation and 200 mg/kg cyclophosphamide. Antibody-mediated in vivo NK cell depletion was applied to demonstrate the NK cell’s role.

**Results:**

Administration of 2.5 instead of 5 million alloreactive NK cells significantly reduced PFS, evidencing dose responsiveness. Compared to MHC-matched receivers of subcutaneous 4T1, fewer MHC-mismatched mice developed tumors, which was due to NK cell alloreactivity because in vivo NK cell depletion facilitated tumor growth. Application of low-dose chemo-irradiation increased plasma levels of NK cell-activating cytokines, NK cell activity and enhanced NK cell-dependent elimination of subcutaneous tumors. Intravenously injected 4T1 was eliminated by alloreactive NK cells in MHC-mismatched recipients without the need for chemo-irradiation.

**Conclusions:**

Bone marrow is a suitable source of sufficient alloreactive NK cells for the cure of 4T1 breast cancer. These results prompt clinical exploration of bone marrow transplantation from NK-alloreactive MHC-mismatched donors in patients with metastasized breast cancer.

## Introduction

Mice with 4T1 breast cancer can be cured by transfer of 5 million alloreactive NK cells after a non-myeloablative dose of total body irradiation and cyclophosphamide (“chemo-irradiation”) [1]. NK cell alloreactivity is present when tumor cells do not express the appropriate major histocompatibility (MHC) alleles for one or more inhibitory receptors of donor NK cells (i.e., “missing self” concept [2]), a situation that typically may occur when using MHC-mismatched donors. Similar anti-cancer effects exerted by transferred alloreactive NK cells have been observed in mouse models for murine [3–6] and human acute myeloid leukemia [7], and for human multiple myeloma [8]. Indications that alloreactive NK cells may be able to kill solid non-hematological tumor tissue comes from a preclinical study where it had been demonstrated that freshly isolated solid tumor tissue is only killed by NK cells from alloreactive and not from non-alloreactive donors [9]. Unequivocal evidence that transfer of alloreactive NK cells exerts an anti-cancer effect in patients does not exist, either because the chemotherapy and/or irradiation applied before the administration may have resulted in increased progression-free survival or because transferred NK cells are not, transiently or only in limited numbers detectable in recipients [10]. It is for this reason that many efforts are currently performed to produce large amounts of NK cells for clinical application, and the success of these approaches is yet to be awaited. As a dose–response relation of the number of transferred alloreactive NK cells and the anti-4T1 breast cancer effect had not been demonstrated yet, we wanted to proof this formally in the current study.

An alternative and clinically applicable source for alloreactive NK cells are MHC-mismatched hematopoietic cells. The advantages of this source are the guaranteed and continuous production of NK cells that are alloreactive toward the patient in case the patient does not express one or more ligands for NK cell inhibitory receptors that are present in the donor. In this setting, donor NK cells mature under the influence of the MHC of the donor’s hematopoietic system which directs the licensing of all NK cells that bear inhibitory receptors for self-MHC resulting in NK cell alloreactivity toward tumor cells of patients that lack the appropriate MHC alleles [11–13]. The possible benefit of this treatment is not hypothetical, as results of retrospective clinical studies show that relapse rates in patients with acute myeloid leukemia after MHC-mismatched hematopoietic stem cell transplantation (HSCT) from NK-alloreactive donors are lower compared to the results with non-NK-alloreactive donors under the condition of a low incidence of acute graft-versus-host disease (GVHD) [14–19]. MHC-mismatched HSCT was, however, until recently a very risky procedure due to a high treatment-related mortality from opportunistic infections due to a prolonged T cell deficiency state when deep T cell depletion was applied to prevent GVHD [15, 20, 21]. However, the now widely used application of post-HSCT cyclophosphamide (PT-CY) prevents prolonged T cell deficiency, high infection rates, and GVHD, and this has made MHC-mismatched HSCT a safe and feasible procedure [22, 23]. NK-alloreactive MHC-mismatched HSCT has never been tested for patients with breast cancer or any other type of non-hematological cancer, and hence we wanted to study the curative potential of alloreactive NK cells that had matured in 4T1 breast cancer-bearing mice. As 4T1 is a rapidly growing tumor and because it takes several months for NK cells to mature and become fully functional after HSCT [13, 24], we employed a surrogate model to mimic treatment of breast cancer-bearing mice with HSCT from a NK-alloreactive MHC-mismatched donor. In this model, MHC-mismatched mice that are NK alloreactive toward 4T1 breast cancer were injected either subcutaneously (s.c.) or intravenously (i.v). with 4T1 cells representing primary and metastasized disease, respectively, and followed for several weeks for tumor development. In some experiments, a non-myeloablative dose of cyclophosphamide and total body irradiation were applied to study if these would increase NK cell-activating cytokine plasma levels, NK cell activation, and the anti-tumor effect, and in vivo NK cell depletion by antibodies was used to demonstrate the indispensability of NK cells for the anti-tumor effect.

## Materials and methods

### Cells, animals, and tumor models

4T1 breast cancer cell line of Balb/cfC3H origin [25] was cultured in RPMI1640. Harvest was after 2 min trypsinization, and 5 × 10^4^ viable cells were injected either s.c. or i.v. Balb/c (H-2^d^), C57BL/6 (“B6,” H-2^b^), (CBA × C57Bl/6)F1 (“B6CBAF1,” H-2^b/k^) and (Balb/c × C57Bl/6)F1 (“CB6F1,” H-2^b/d^) mice were from Harlan Laboratories (Horst, the Netherlands) and housed under specified pathogen-free conditions. Mice were in follow-up for at least 100 days after tumor induction and underwent standard autopsy for the presence of lung and liver metastases which we never found in mice that were cured from their s.c. tumors. Balb/c mice that succumbed from breast cancer regularly had metastases in lungs and liver, while this was occasionally the case for B6CBAF1 mice with s.c. tumors. All B6CBAF1 mice that died from i.v. injected 4T1 tumor cells had pulmonary metastases.

For the study of a dose–response effect of transferred NK cells, 4T1-bearing Balb/c mice were used. In the second set of experiments where the anti-tumor effect of NK cells that had endogenously matured from bone marrow in the tumor-bearing host, we chose a model where MHC-mismatched B6CBAF1 mice served as recipients of 4T1 breast cancer cells instead of transplanting MHC-mismatched HSC into Balb/c 4T1-bearing mice. This was because the rapid growth of the 4T1 breast cancer cells, even after chemo-irradiation, would not allow sufficient time for the donor-derived alloreactive NK cells to become mature and active. S.c. injected 4T1 was used as a model for localized breast cancer, while i.v. injection of 4T1 cells mimics the process of metastasis.

### Chemo-irradiation

Chemo-irradiation was performed by 2× 2 Gy TBI (PHILIPS X-ray unit, 225 kV, 10 mA, dose-rate 66 cGy/min) at 8 and 9 days after tumor induction combined with 200 mg/kg cyclophosphamide (Baxter Oncology GmbH, Halle, Germany) at day 9. This conditioning is non-myeloablative in mice [26].

### NK cell transfer

NK cell-enriched spleen cell batches were prepared from single cell suspensions from the spleens of donor mice by MACS negative selection (Miltenyi Biotec B.V., Utrecht, the Netherlands); a typical NK cell dose of 5 × 10^6^ NK cells contained approximately 0.06 × 10^6^ T cells. We made use of a difference in frequency of NK cells with alloreactive activity toward H-2^d^ target cells of various strains of mice as dictated by their H-2 type. Whereas all NK cells of B6 (H-2^b^) and B6CBAF1 (H-2^b/k^) mice are alloreactive toward H-2^d^ target cells (i.e., “full-alloreactive”), only half of the NK cells in CB6F1 (H-2^b/d^) mice are (i.e., “half-alloreactive”). This difference has been shown to translate in faster in vivo elimination of H-2^d^-positive target cells by full-alloreactive NK cells than by half-alloreactive NK cells [1, 27, 28]. Besides, CB6F1 T cells are tolerant to Balb/c tissue and are therefore not able to act as alloimmune effector cells.

### In vivo NK cell depletion

In vivo NK cell depletion was performed in two consecutive experiments by intraperitoneal (i.p.) injection with either anti-AsialoGM1 or anti-NK1.1. We chose for using both antibodies to be sure that the effect of antibody administration on the anti-tumor effect could most surely be attributed to the depletion of NK cells only, because subsets of T cells express AsialoGM1 and many NKT cells express NK1.1 [29, 30]. 200 µl of mouse-specific polyclonal rabbit anti-AsialoGM-1 (Wako Pure Chemical Industries) [31] was administered intraperitoneally at days 0, 5, and 10 after tumor induction. In the next experiment, 500 µg anti-NK1.1 (PK136, BD Pharmingen) [29, 32] was administered intraperitoneally at day 0, 5, and then every other 5 days until the death of the mice or the end of the experiment, because we realized from the results of the experiment with anti-AsialoGM1 that this should have been administered during the whole experiment too. The NK cell depletion efficacy had been previously checked in three mice for each antibody by measuring the NK cell content in blood and spleen just before the time of second administration of either anti-AsialoGM1 or anti-NK1.1; NK cell depletion amounted approx. 1 log in all.

### Plasma NK cell-activating cytokine level measurements

Plasma was prepared from blood drawn from just priorly euthanatized mice. IL-2, IL-15, IL-18, and IL-21 plasma levels were determined by standard ELISA (R&D Systems).

### Flowcytometry

All flowcytometry analyses were performed using a BD FACS CantoII flow cytometer using BD DIVA software. In vivo NK cell depletion efficiency was by staining spleen single-cell suspensions with the mouse-specific antibodies CD3e PerCP, and CD49b APC after incubation with NMS. NK cell activation was determined with antibodies specific for CD69, CD107a, TRAIL, and FasL. 7-AAD or Pi was for excluding non-viable cells, and all flowcytometry analyses were exclusively on viable cells. All monoclonal antibodies and 7-AAD were from BD Biosciences, and Pi was from Invitrogen.

### Ethical approval

The local animal ethical committee had approved all the mouse experiments.

### Statistics

Survival curves were composed using the Kaplan–Meier method and compared with the Mantel–Cox log-rank test. Differences in cytokine levels and expression of NK cell activation markers after chemo-irradiation were compared with non-chemo-irradiated mice using the two-tailed Wilcoxon’s signed-rank sum test. Values are presented as mean ± SEM. In all cases, differences were considered statistically significant when probability (*p*) values were less than 0.05.

## Results

### High numbers of alloreactive NK cells are required to cure 4T1 breast cancer

**Fig. 1:**
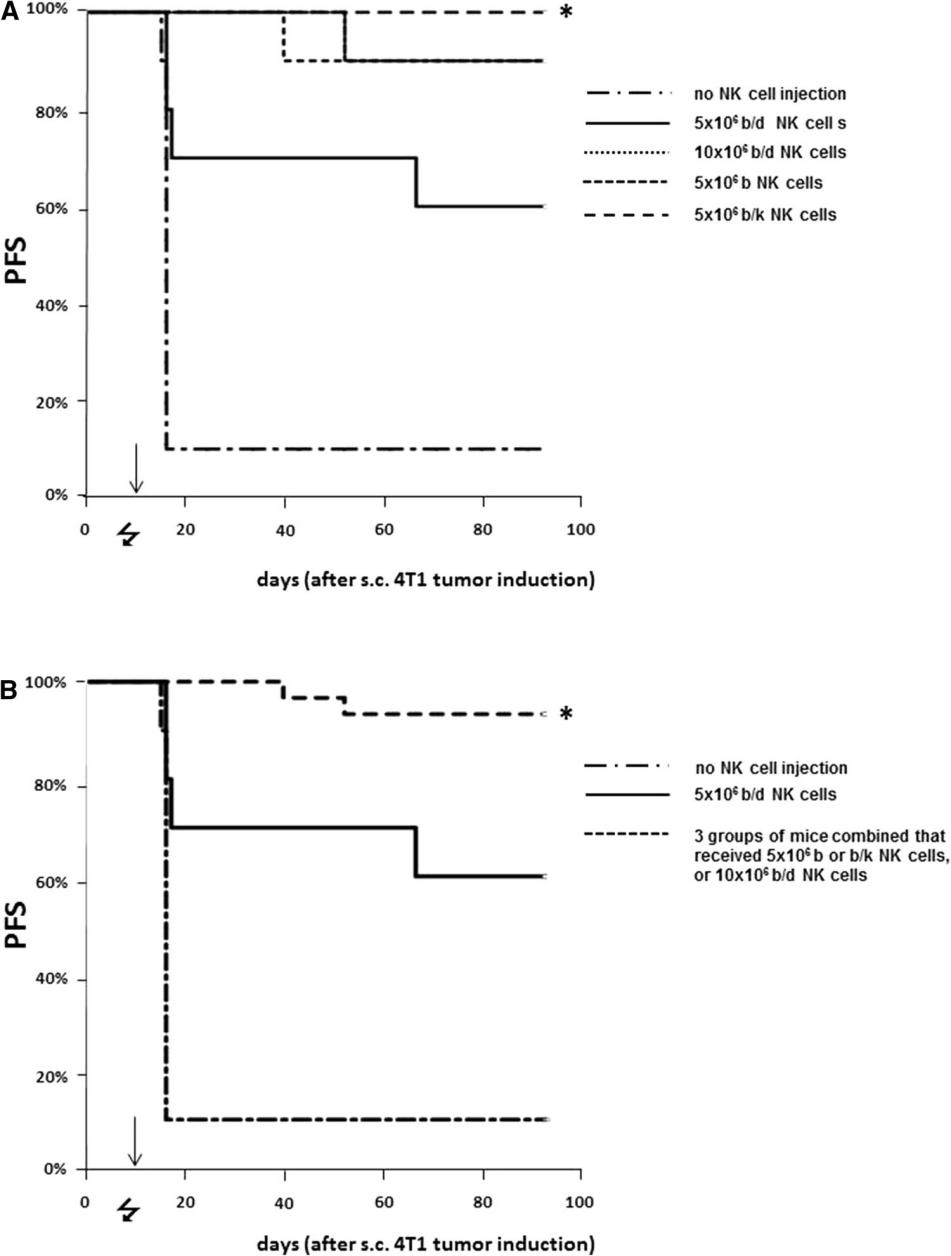
Improved PFS is alloreactive NK cell dose dependent. Shown are PFS curves of one experiment where Balb/c mice were injected s.c. with 4T1 breast cancer at day 0 and either left untreated or treated with chemo-irradiation at days 8 and 9 (indicated by the  symbol) followed by transfer of NK cells from various mouse strains with different numbers of alloreactive NK cells toward the Balb/c-type tumor at day 10 (indicated by the *arrow*, 10 mice per group; “b/d” (CB6F1) mice have half the number of alloreactive NK cells than “b” (B6) or “b/k” (B6CBAF1) mice). **a** The PFS of each treatment group separately and **b** The PFS of recipients of equal doses of alloreactive NK cells grouped together. *Statistical significant difference (*p* < 0.01) of the PFS of mice treated with 5 × 10^6^ b/k NK cells compared with PFS of mice without NK cell treatment as well as with the groups that received the same 2× higher number of alloreactive NK cells (i.e., 5 × 10^6^ b or b/k NK cells or 10 × 10^6^ b/k NK cells) taken together

To demonstrate dose dependency of the anti-4T1 breast cancer effect by transferred alloreactive NK cells, 4T1-bearing Balb/c mice were treated with chemo-irradiation at 8 and 9 days after 4T1 injection followed by i.v. injection of 5 million NK cells from various donors that differed with respect to the percentage of NK cell alloreactivity toward Balb/c-type cells (as described in "Materials and methods" section). Long-lasting breast cancer-free survival resulted in the vast majority (19 out of 20) of mice that received 5 million NK cells from full-alloreactive B6 or B6CBAF1 donors or 10 million NK cells from half-alloreactive CB6F1 mice (9 out of 10), while 90% of the untreated mice died from tumor progression (*p* < 0.001 for each treatment group, Fig. 1a). Although the administration of 5 million half-alloreactive CB6F1 NK cells (equivalent to 2.5 million fully alloreactive NK cells) resulted in cure in 6 out of 10 mice (*p* < 0.01 compared to untreated mice), its efficacy was statistically significantly lower than in the three groups that received 5 million fully alloreactive NK cells (i.e., either 5 million full-alloreactive NK cells from either B6 or B6CBAF1 donors or 10 million half-alloreactive NK cells from CB6F1 donors (*p* < 0.01 when these three groups are compared with the PFS of mice that received 5 million half-alloreactive NK cells from CB6F1 donors, Fig. 1b). The results of administration of 10 million half-alloreactive CB6F1 NK cells also demonstrate that effective alloreactive NK cell therapy does not require T cell alloreactivity, as CB6F1 T cells are tolerant toward Balb/c-MHC type.

Our results show that fairly high numbers of alloreactive NK cells are required for an effective anti-tumor response. While the procedures for NK cell expansion that reliably produces this high number of NK cells for clinical application are improving over the last years, only a few look promising but are still in the experimental phase. We therefore studied if the number of NK cells that had been produced and matured in MHC-mismatched tumor-receiving mice is sufficient to eliminate fast-growing 4T1 tumors, as a surrogate model for donor HSCT.

### Alloreactive NK cells that had matured in the 4T1-bearing host optimally eliminate MHC-mismatched s.c. breast cancer after a low dose of chemo-radiotherapy

S.c. injection of full-MHC-mismatched Balb/c-type 4T1 breast cancer cells in B6CBAF1 recipients surprisingly induced tumors in many mice (Table 1, exp. nos. 1–4, “only s.c. 4T1 tumor induction” groups). When these tumor-bearing mice were subsequently treated with the same low-dose chemo-irradiation as used in the above and previously described [1] transplantation experiments at 8 and 9 days after 4T1 tumor induction, tumor growth was permanently arrested in the vast majority of mice (Table 1, exp. nos. 2–4, “chemo-irradiation” groups). As we hypothesized, but not yet had proven, that the observed anti-tumor effect induced by chemo-irradiation relies on a NK cell alloimmune response toward the tumor, we then tested if in vivo NK cell depletion by antibodies would impede the anti-tumor effect.

**Table 1:** Low-dose chemo-irradiation enhances the anti-tumor effect of alloreactive NK cells that had developed and matured in the tumor-bearing fully MHC-mismatched host

Exp. no.	Treatment group (all had s.c. 4T1 breast cancer)	Palpable tumor incidence			
		At day 8	*p* value	At the end of the experiment	*p* value
1.	No further treatment	0/12		10/12	
2.	No further treatment	0/12	–	10/12	<0.01
	Chemo-irradiation	0/12	Reference	2/12	Reference
3.	No further treatment	6/16	–	11/16	0.01
	Chemo-irradiation	5/16	Reference	3/16	Reference
	Anti-AsialoGM1 NK cell depletion at days 0, 5, and 10 and chemo-irradiation	10/16	0.12	8/16	0.14
4.	No further treatment	4/16	–	9/16	0.07
	Chemo-irradiation	4/16	Reference	3/16	Reference
	Anti-NK1.1 NK cell depletion from day 0 and chemo-irradiation	12/16	0.01	13/16	<0.01

Palpable s.c. 4T1 breast cancer incidence as measured at day 8 (i.e., the day of onset of chemo-irradiation in the appropriate groups) and at the end of the experiment (130 days in experiments 1–3, 100 days in experiment 4)

**Fig. 2:**
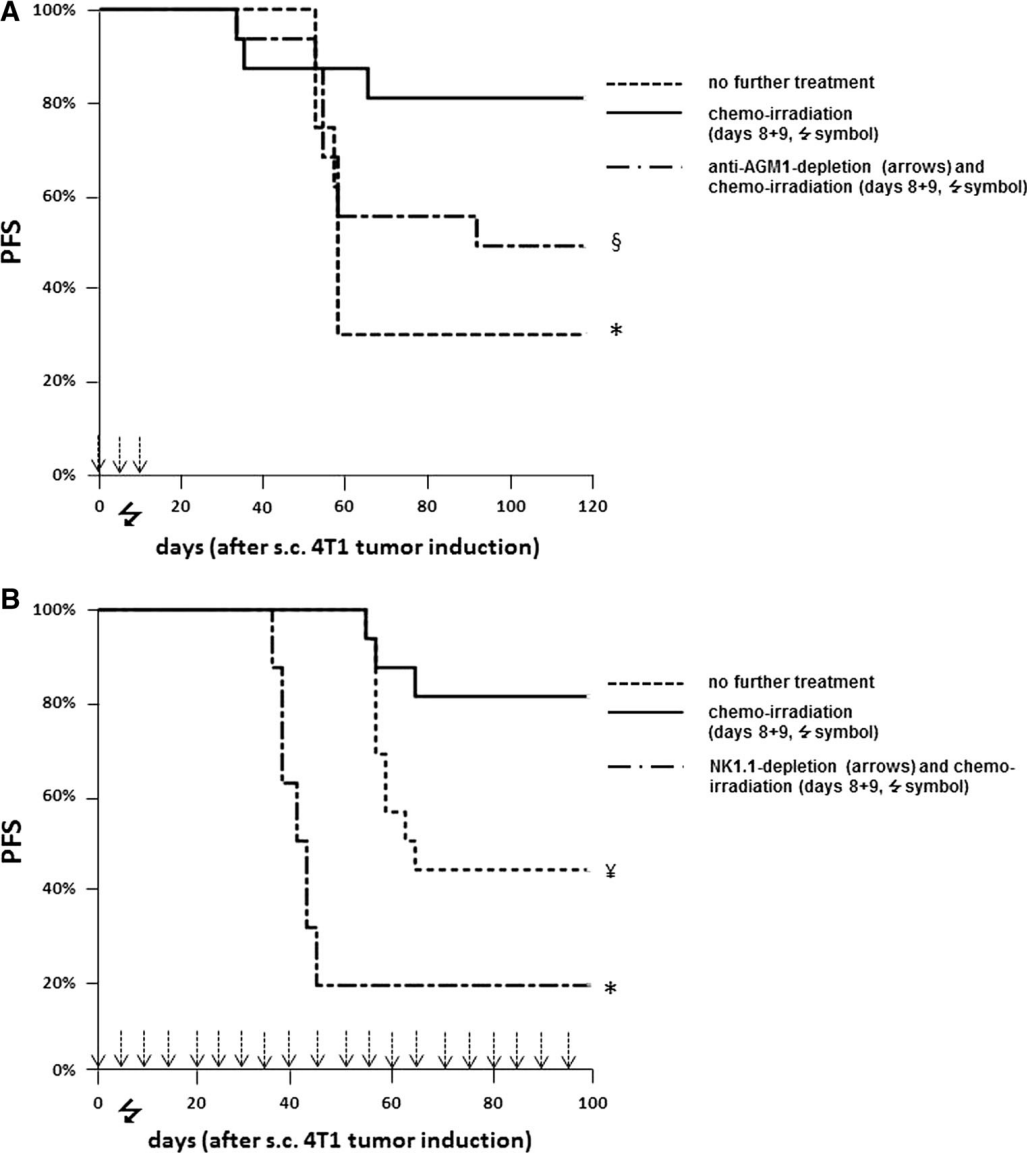
NK cells mediate s.c. injected 4T1 breast cancer elimination in MHC-mismatched hosts after chemo-irradiation. PFS curves of two separate experiments where B6CBAF1 mice were s.c. injected with 4T1 breast cancer cells at day 0 followed by either no further treatment (*n* = 16), chemo-irradiation at days 8 and 9 (*n* = 16, indicated by the  symbol), AsialoGM1 depletion [**a** *n* = 16, anti-AGM1 applied (*arrows*)during the first 2 weeks, exp. A] or NK1.1 depletion [**b** *n* = 16, anti-NK1.1 applied (*arrows*) during whole experiment], in both experiments combined with chemo-irradiation at days 8 and 9. All events were breast cancer-related deaths. Statistically significant differences or trends of PFS compared to the chemo-irradiation groups are indicated by *p < 0.01, ^¥^ *p* = 0.03, and ^§^ *p* = 0.09

In the first experiment, in vivo NK cell depletion was with anti-AGM1 administration during the first 2 weeks after tumor induction (Table 1, exp. no. 3). Depletion of AsialoGM1-positive cells resulted in an accelerated tumor growth in the first week after tumor induction compared to the untreated group: 10 of the 16 anti-AsialoGM1-treated mice had developed a palpable tumor at day 8 (being the first time point of chemo-irradiation) with tumor sizes ranging from 37 to 85 mm^3^, compared to 6 of the 16 in the untreated group where tumor sizes ranged from 5 to 10 mm^3^ (*p* < 0.01 for tumor size) (Table 1, exp. no. 3). In the anti-AsialoGM1-treated group, initially all palpable tumors disappeared after chemo-irradiation, but then reappeared and grew in 8 out of the 16 (7 had s.c. tumors, 1 deceased from pulmonary metastases). The mice treated with chemo-irradiation without anti-AGM1 treatment had similar tumor incidence and tumor size as in the untreated group at day 8, but in only 3 out of 16 progressive s.c. tumors (re-)appeared after chemo-irradiation, while 11 of the 16 untreated mice died from s.c. progressive tumor growth (*n* = 7) or from pulmonary metastases (*n* = 4). The PFS was significantly better in the chemo-irradiated group compared to the untreated ones (Fig. 2a, *p* < 0.01), while there was a trend for inferior PFS in the chemo-irradiated and anti-AGM1-treated mice compared to those treated with chemo-irradiation only (Fig. 2a, *p* = 0.09). It was felt that the in vivo NK cell depletion in this experiment might had lasted too short to unequivocally demonstrate the role of NK cells in the chemo-irradiation-induced anti-tumor effect.

In the second experiment with in vivo NK cell depletion, anti-NK1.1 was administered throughout the whole experiment (Table 1, exp. no. 4). Similar to the former NK cell depletion experiment, the incidence of 4T1 breast cancer in the first week after tumor induction was statistically significantly higher than in the “no further treatment” group (Table 1, exp. no. 4). As in the former depletion experiment, tumors shrunk shortly after chemo-irradiation but later reappeared in all but one (Table 1, exp. no. 4). In the chemo-irradiated mice without anti-NK1.1 treatment, only three died from tumor progression, while nine mice in the untreated group succumbed from progressive breast cancer. PFS of the chemo-irradiated and NK1.1-positive cell-depleted group was inferior to the group of mice treated with chemo-irradiation only (Fig. 2b, *p* < 0.01) and to the “no further treatment” group (Fig. 2b, *p* < 0.01), and PFS of chemo-irradiated mice was better than that of the untreated mice (*p* = 0.03).

Taken together, these results demonstrate the indispensable role of NK cells both in delaying tumor progression and in chemo-radiation-induced eradication of s.c. 4T1 breast cancer in fully MHC-mismatched B6CBAF1 mice.

### Low-dose CY+TBI results in increased plasma levels of NK cell-activating cytokines as well as in NK cell activation

**Fig. 3:**
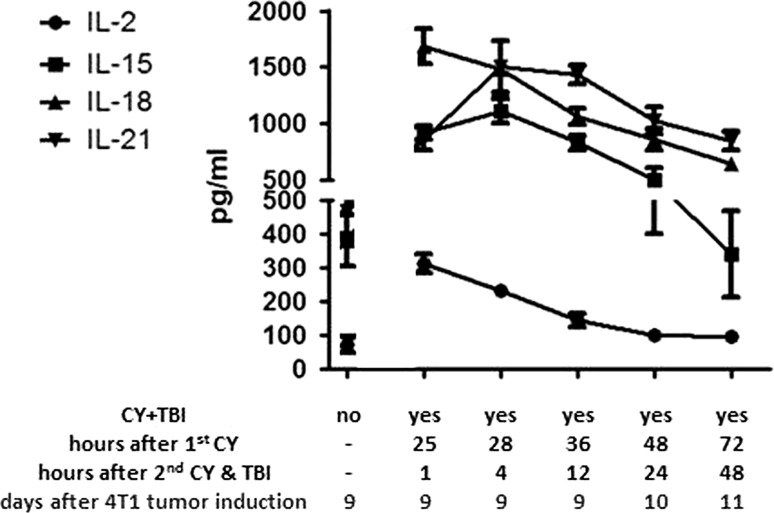
Chemo-irradiation leads to increased plasma levels of NK cell-activating cytokines. Plasma levels of IL-2, IL-15, IL-18, and IL-21 were measured at different time points after treatment of 4T1 tumor-bearing B6CBAF1 mice with CY+TBI, as indicated. Per time point the median plasma level, and SEM are shown of five mice per time point. The increases in plasma levels were highly statistically significant (*p* < 0.001) compared to non-chemo-irradiated mice for all four measured cytokines at the first three time points and statistically significant (*p* < 0.01) for IL-15, IL-18, and IL-21 at the two last time points

**Fig. 4:**
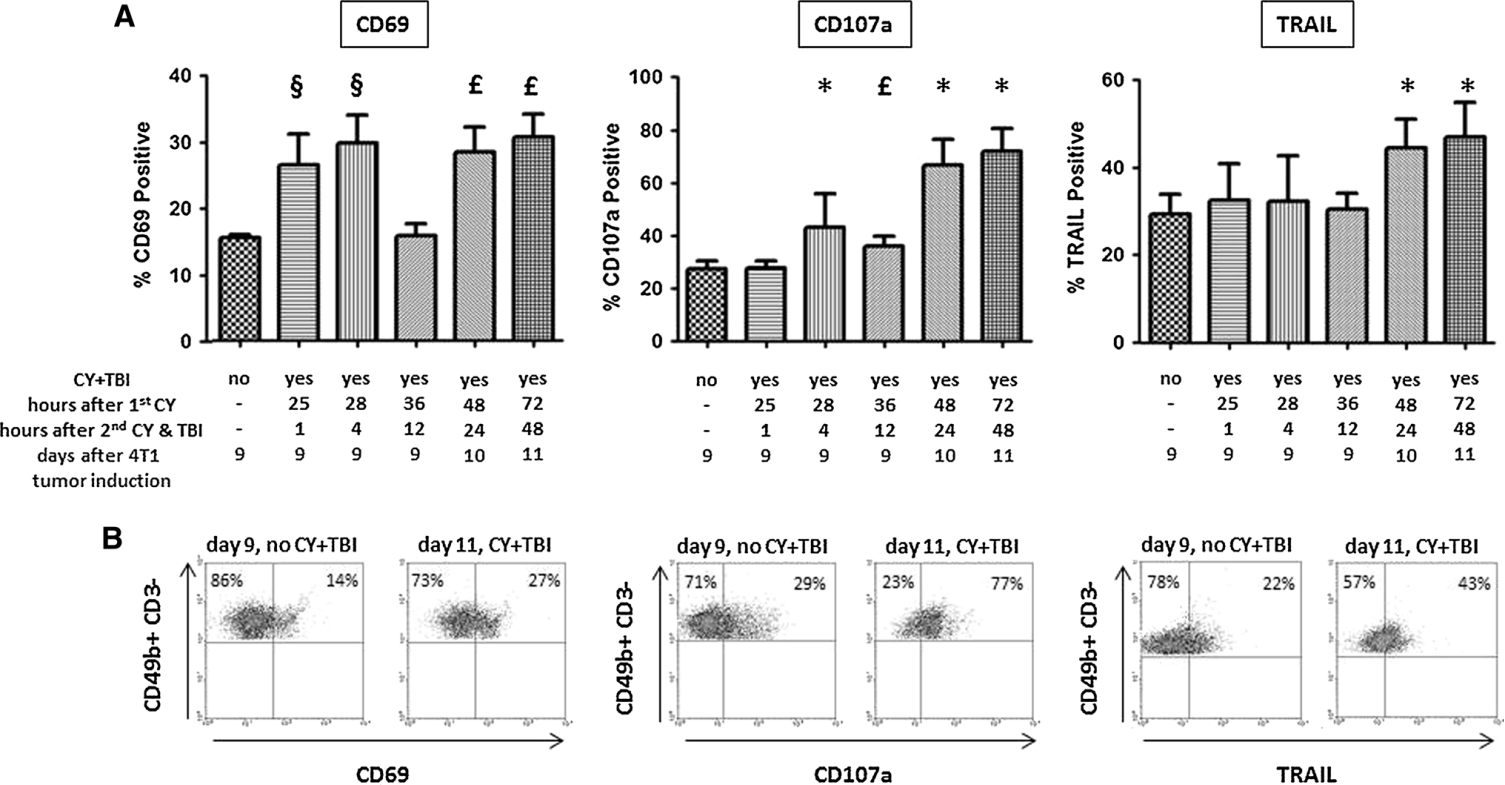
Chemo-irradiation results in activation of NK cells. NK cell activation was measured by increased CD69 and TRAIL expression and by increased degranulation (cell surface CD107a expression) of splenic NK cells at different time points after treatment of 4T1 tumor-bearing B6CBAF1 mice with CY+TBI, as indicated. NK cells were identified as CD3-negative and CD49b-positive lymphocytes. **a** The percentages (and SEM) of NK cells positive for CD69, CD107a, and TRAIL of five mice per time point: 9 days after 4T1 tumor induction without treatment with CY+TBI, and at successive time points after CY+TBI. ^§^ *p* < 0.05, ^£^ *p* < 0.01, *p < 0.001 compared with 9 days after 4T1 tumor induction without chemo-irradiation. **b** Examples of FACS plots of CD69, CD107a, and TRAIL expression of splenic NK cells of individual 4T1 tumor-bearing mice at the time points indicated

Plasma levels of the NK cell-activating cytokines IL-2, IL-15, IL-18, and IL-21 [33] increased substantially within the first 2 days after CY+TBI compared to their plasma levels in 4T1 tumor-bearing mice not subjected to chemo-irradiation (Fig. 3). Thereafter, cytokine plasma concentrations steadily decreased. To investigate if this increased plasma levels of cytokines is accompanied with NK cell activation, the activation status of splenic NK cells, as measured by their CD69 [34], TRAIL, and FasL expression [35] and degranulation status (by surface CD107a expression) [36], was also determined at the same time points (Fig. 4). For CD69, we found statistically significant increased expression already at the first time point, i.e., 1 h after the 2nd cyclophosphamide dose and TBI, that persisted, with a short lasting drop, till the last measurement point at 48 h after the 2nd cyclophosphamide dose and TBI. A statistically significant increase in the percentage of degranulating NK cells and in TRAIL expression was evident from 24 h after the 2nd cyclophosphamide dose and TBI. FasL expression had doubled on all NK cells at all test points (*p* < 0.001, data not shown).

### Low-dose CY+TBI is not required for NK cell-mediated in vivo elimination of i.v. injected MHC-mismatched breast cancer cells

As i.v. injection of tumor cells mimics the process of blood-born pulmonary metastasis, we tested whether alloreactive NK cells are able to prevent metastasis. For this purpose, we again used the fully MHC-mismatched B6CBAF1 recipient model. I.v. injection of 4T1 breast cancer in half-MHC-matched CB6F1 recipients resulted in pulmonary metastases in all mice, while none of the B6CBAF1 mice developed any pulmonary distress during 130 days of follow-up nor lung metastases at autopsy (Table 2, exp. no. 5).

**Table 2:** Alloreactive NK cells prevent pulmonary metastasis of i.v. injected 4T1 breast cancer cells in the fully MHC-mismatched host

Exp. no.	Treatment group (all had i.v. 4T1 tumor injection)	Recipient mouse strain	Mortality from pulmonary metastases	*p* value
5.	No further treatment	CB6F1	10/10	Reference
	No further treatment	B6CBAF1	0/11	<0.01
6.	No further treatment	B6CBAF1	2/16	Reference
	Anti-AsialoGM1 NK cell depletion at days 0, 5, and 10	B6CBAF1	8/16	0.06
7.	No further treatment	B6CBAF1	3/16	Reference
	Anti-NK1.1 NK cell depletion from day 0 until the end of the experiment	B6CBAF1	12/15	<0.01

Pulmonary metastases were evident by development of severe pulmonary distress requiring sacrifice. Follow-up time was 130 days after 4T1 i.v. injection in exp. nos. 5 and 6, and 100 days in exp. no. 7

**Fig. 5:**
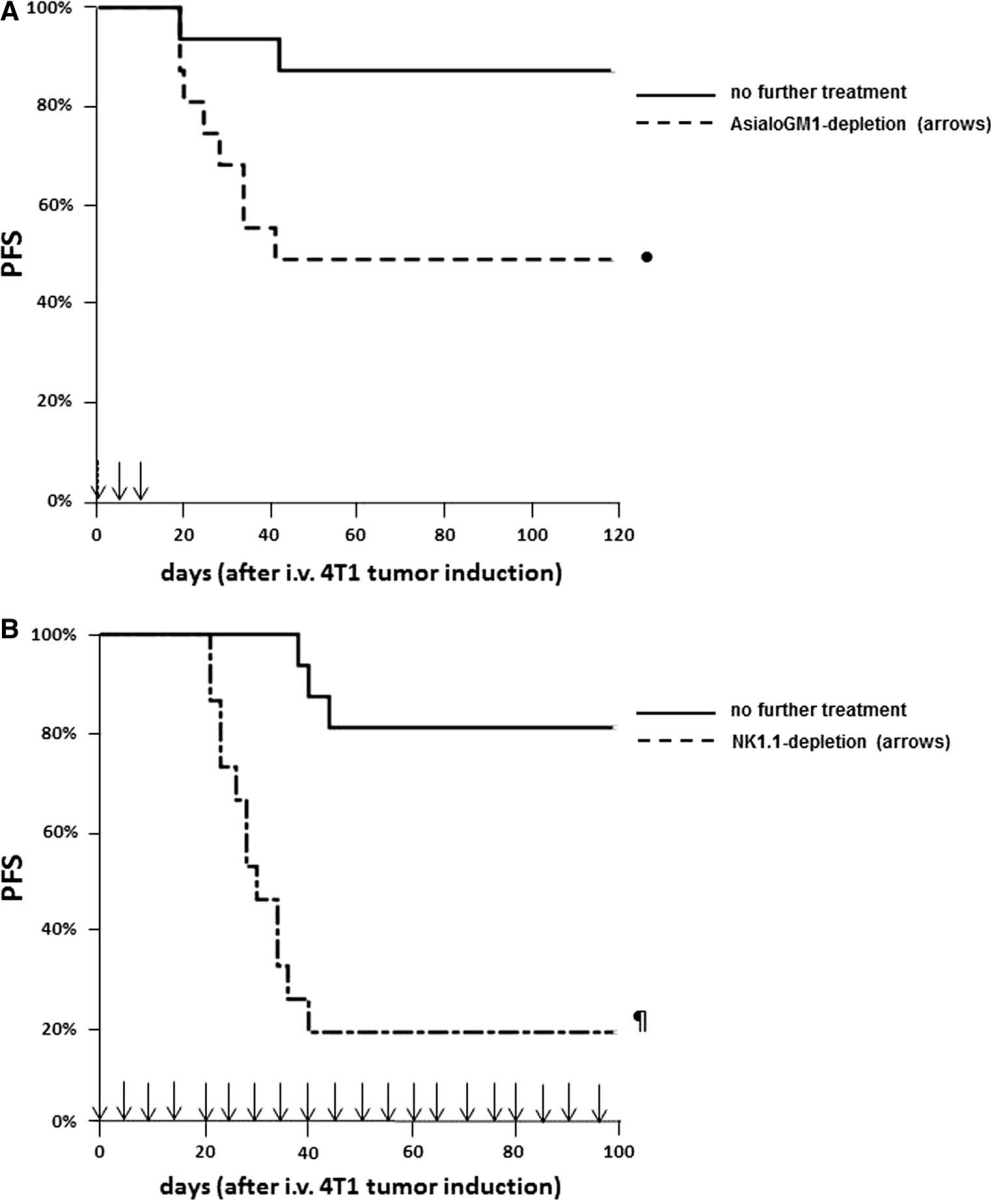
NK cells mediate i.v. injected 4T1 breast cancer elimination in MHC-mismatched hosts. PFS curves of two separate experiments where B6CBAF1 mice were injected with 4T1 breast cancer cells i.v. at day 0 followed by either no further treatment (*n* = 16) or AsialoGM1 depletion [**a** *n* = 16, anti-AGM1 applied (*arrows*) during the first 2 weeks] or NK1.1 depletion [**b** *n* = 15, anti-NK1.1 applied (*arrows*) during whole experiment]. All events were breast cancer-related deaths. Statistically significant differences or trends of PFS compared to the “no further treatment” groups are indicated by ^¶^ *p* < 0.001 and ^**•**^ *p* = 0.02

We then applied in vivo NK cell depletion by anti-AGM1 or anti-NK1.1 to test whether the prevention of pulmonary metastasis in the MHC-mismatched B6CBAF1 mice resulted from NK cell activity. Short-term AGM1-postive cell depletion almost statistically significantly increased mortality (Table 2, exp. no. 6) and statistically significantly decreased PFS compared to untreated tumor-injected mice (Fig. 5a, *p* = 0.02; all deceased mice had lung metastases at autopsy). Similarly, administration of anti-NK1.1 every other 5 days from the time of i.v. tumor injection until the day of sacrifice or the end of the observation time resulted in a statistically significant decreased survival (Table 2, exp. no. 7) and decreased PFS (Fig. 5b, *p* < 0.001). These data demonstrate that alloreactive NK cells are a prerequisite for elimination of i.v. injected H-2-mismatched 4T1 breast cancer cells.

## Discussion

In this study, we demonstrated a dose–response relation between adoptively transferred NK cells from NK-alloreactive donors and the anti-tumor effect as well as the dispensability of alloreactive T cells in the 4T1 mouse breast cancer model. The human equivalent of the minimally required number of full-alloreactive NK cells per mouse (5 million for a mouse weighing 20 g amounts 0.25 × 10^9^/kg) would be 18.75 × 10^9^ for a patient weighing 75 kg. This number can never be harvested from a donor in a single procedure and necessitates in vitro NK cell expansion. Each individual mouse and man bears NK cell subsets expressing different inhibitory and activating receptors. Two preconditions determine if a given donor NK cell is alloreactive: (1) membrane expression of iKIR specific for a ligand that is present in the donor and absent in the patient (i.e., certain MHC class I alleles) and (2) no NKG2A expression (inhibitory receptor binding ubiquitously expressed HLA-E that is not subject to allelic differences with respect to binding to NKG2A). Additional prerequisites for successful clinical application of expanded NK cells are sufficient numbers and absence of donor T cells causing severe GVHD. At present, the vast majority of the laboratories working on clinical grade expansion of NK cells do not unequivocally demonstrate that their NK cell products meet all four prerequisites [37–49]. Only recently a report was published on a successful though laborious expansion procedure in the presence of membrane-bound IL-21, which resulted in preserved KIR expression and NKG2A absence [50]. Feasibility of the clinical application of this NK cell product is yet to be awaited. It remains, in general, also to be seen, if a single administration of alloreactive NK cells which results in only a transient engraftment is as effective in patients like in our mouse model. This then justifies the exploration of alternative ways to apply NK cells, especially when resulting in the permanent presence of alloreactive NK cells in the patient.

One such an alternative strategy is to apply HSCT from NK-alloreactive MHC-mismatched donors as a permanent source of NK cells, which is nowadays a fairly safe procedure when PT-CY is applied [22, 23]. Reports on MHC-mismatched HSCT from NK-alloreactive donors all point toward a benefit with respect to leukemia-free survival in settings with a low incidence of acute GVHD [15–17, 51, 52], and the role of alloreactive NK cells in this setting is underscored by the fact that these cells are fully functional within a few months after HSCT [11–13, 53, 54]. Advantages of NK cell therapy by MHC-mismatched HSCT are that it results in a permanent production of alloreactive NK cells, as the latter quality hinges on the origin of the hematopoietic cells (in this case the donor’s) among which they mature [11–13]. In contrast, adoptively transferred NK cells will probably lose their alloreactive quality within days due to exposure to the MHC-disparate patient’s hematopoietic system [55–57].

In this study, treatment with NK-alloreactive MHC-mismatched HSCT of a breast cancer patient was mimicked using a MHC-mismatched host as recipient of 4T1 breast cancer, because the 4T1’s far more rapid tumor growth than human breast cancer results in death weeks before the donor bone marrow would have produced enough functional NK cells. We demonstrated that both s.c. and i.v. injected 4T1 breast cancer is eradicated by AGM1- and NK1.1-positive cells. As only NK cells share both characteristics [58, 59] and because NK cells alone are sufficient for cure in the NK cell transfer model [1] [and this manuscript], it is apparent that the anti-tumor effect resulted from the MHC-mismatched host’s activated NK cells.

The elimination of i.v. injected tumor cells (including tumorigenic cells) by the NK cells of the NK-alloreactive recipient was efficient and did not require further treatment. This indicates that breast cancer metastasis in patients after NK-alloreactive MHC-mismatched HSCT may be prevented by the circulating donor-derived NK cells. For extra-vascular tumor sites, additional treatment with low-dose chemo-irradiation seems warranted for NK cell-mediated elimination. This indicates that chemo-irradiation may be required in patients with metastasized breast cancer when functional donor-derived NK cells have been produced several months after NK-alloreactive MHC-mismatched HSCT. During the time of NK cell repertoire constitution, tumor growth should be prevented by the application of myeloablative conditioning before MHC-mismatched HCSCT [60] which has shown to be feasible even in heavily pretreated patients [61], and by hormonal and/or anti-HER2neu therapies when appropriate.

Puzzling is that adoptive transfer of sufficient numbers of CB6F1 semi-MHC-mismatched NK cells cures Balb/c mice from 4T1 breast cancer, while s.c. and i.v. injection of 4T1 in CB6F1 mice invariably results in high tumor-induced mortality even when chemo-irradiation is applied in contrast to the results in fully MHC-mismatched B6CBAF1 mice. This discrepancy may be a matter of the number of actual alloreactive NK cells toward the Balb/c-type tumor. The fact that half-MHC-mismatched HSCT from NK-alloreactive donors is very effective in preventing leukemia relapse in patients [15–17, 51, 52] brings promise that the number of alloreactive NK cells several months after half-MHC-mismatched HSCT will also be sufficient for the elimination of residual breast cancer after the conditioning before HSCT and the subsequent application of chemo-irradiation.

Various mechanisms may contribute to the potentiation by chemo-irradiation of the NK cell-mediated anti-tumor effect. One may be related to the ascertained NK cell activation either by the observed increase in plasma levels of pro-inflammatory and NK cell-activating cytokines, possibly related to the chemo-irradiation-induced reduction in lymphocyte number [62–65], and/or by translocation of commensal gut flora and LPS [63]. Although the number of NK cells also decreases after chemo-irradiation [63], their numbers may still have remained sufficiently high to eradicate 4T1 breast cancer upon their cytokine-induced activation, analogous to the observation that i.v. injected MHC-mismatched hematopoietic stem cells are rejected by host NK cells even in lethally irradiated mice (the so-called “hybrid resistance model” [66]). Another effect may be a reduction of 4T1 tumor-induced NK cell inhibitory elements like regulatory T cells and myeloid-derived suppressor cells [63, 67–73], similar to its relevance for disease-specific T-cell therapy [74].

What may the implications of our findings be for the treatment of patients with metastasized breast cancer? Adaptive transfer of alloreactive NK cells may at first glance seem the most preferable therapy as it evades the possible risks resulting from haploidentical HSCT like GVHD and infections. The toxicity of adaptive NK cell therapy in human patients is anticipated to be low based on present-day experience, although the numbers of transferred NK cells were not as high as we would aim at based on our dose–response experiment [75–77]. It remains to be seen if the application of higher numbers of alloreactive NK cells resulting in short-term engraftment will be as curative as in our mouse model. Haploidentical HSCT from NK-alloreactive donors and the subsequent application of low-dose chemo-irradiation after NK cell repertoire maturation is then an intriguing and promising treatment approach as it results in the continuous production of alloreactive NK cells in women with metastasized breast cancer. When applicable, it may serve as a platform for subsequent application of low-dose CY+TBI and/or other immune-modulating treatments to enhance the alloreactive NK cell effect.
